# Neurogenic fever due to injury of the hypothalamus in a stroke patient: Case report

**DOI:** 10.1097/MD.0000000000024053

**Published:** 2021-04-02

**Authors:** Sung Ho Jang, You Sung Seo

**Affiliations:** Department of Physical Medicine and Rehabilitation, College of Medicine, Yeungnam University, Namku, Taegu, Republic of Korea.

**Keywords:** diffusion tensor imaging, hypothalamus, intracerebral hemorrhage, intraventricular hemorrhage, neurogenic fever

## Abstract

**Rationale:**

Neurogenic fever is a non-infectious source of fever in a patient with brain injury, especially hypothalamic injury. We report on a stroke patient with neurogenic fever due to injury of hypothalamus, demonstrated by using diffusion tensor imaging (DTI).

**Patient concerns:**

A 28-year-old male patient was admitted to the rehabilitation department of university hospital at 30 months after onset. Brain MRI showed leukomalactic lesions in hypothalamus, bilateral medial temporal lobe, and bilateral basal ganglia. He showed intermittent high body temperature (maximum:39.5°C, range:38.5–39.2°C), but did not show any infection signs upon physical examination or after assessing his white blood cell count and inflammatory enzyme levels such as erythrocyte sedimentation rate and C-reactive protein. In addition, 8 age-matched normal (control) subjects (4 male, mean age: 26.6 years, range: 21–29years) were enrolled in the study.

**Diagnosis:**

Intraventricular hemorrhage and intracerebral hemorrhage in the left basal ganglia.

**Interventions:**

He underwent extraventricular drainage and ventriculoperitoneal shunting for hydrocephalus.

**Outcomes:**

DTI was performed at 30 months after onset, fractional anisotropy (FA) and apparent diffusion coefficient (ADC) values were obtained for hypothalamus. The FA and ADC values of patient were lower and higher, respectively, by more than two standard deviations from control values. Injury of hypothalamus was demonstrated in a stroke patient with neurogenic fever.

**Lessions:**

Our results suggest that evaluation of hypothalamus using DTI would be helpful in patients show unexplained fever following brain injury.

## 1 Introduction

Neurogenic fever is a non-infectious source of fever in a patient with brain injury, especially hypothalamic injury.^[1–3]^ Precise diagnosis of neurogenic fever is clinically important because it is related to a poor outcome; thus, there is a need to undertake differential diagnosis from a fever that originated from infection or inflammatory disease.^[1–3]^

The hypothalamus, a main autonomic center, is involved in regulation of temperature, sleep–wakefulness cycle, and emotional behavior.^[4,5]^ Accurate estimation of the state of the hypothalamus in the live human brain has been limited due to its anatomical characteristics; deep location, and small size.^[4]^ However, the diffusion tensor imaging (DTI) technique has enabled evaluation of the hypothalamus based on DTI parameters.^[6–10]^ Several studies using DTI have reported that injury of the hypothalamus was associated with narcolepsy, hypersomnia, fatigue, and depression in patients with brain injury.^[6–10]^ However, no study on neurogenic fever due to injury of the hypothalamus has been reported.

In the present study, we report on a stroke patient who showed neurogenic fever due to injury of the hypothalamus identified by using DTI.

## 2 Case report

**Figure 1:**
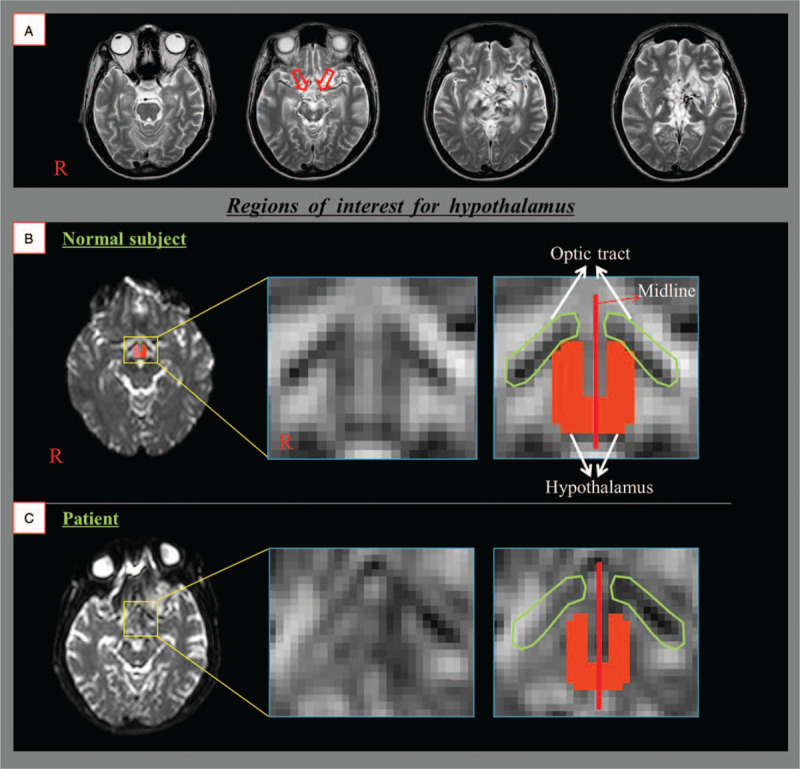
(A) T2-weighted brain magnetic resonance images show leukomalactic lesions in the hypothalamus (arrows), bilateral medial temporal lobe, and bilateral basal ganglia. (B) Regions of interest for the hypothalamus were localized by using the optic tract (anterior boundary), the mammillary body (posterior boundary), and the midline (medial boundary) at the level of the upper midbrain in the patient (C) and a representative normal subject.

A 28-year-old male patient with intraventricular hemorrhage and intracerebral hemorrhage in the left basal ganglia underwent extraventricular drainage and ventriculoperitoneal shunting for hydrocephalus. At 30 months after onset, he was admitted to the rehabilitation department of our university hospital. Brain magnetic resonance imaging (MRI) showed leukomalactic lesions in the hypothalamus, bilateral medial temporal lobe, and bilateral basal ganglia (Fig. 1A). He showed intermittent high body temperature (maximum: 39.5°C, 38.5–39.2°C). However, he did not show any signs of infection upon physical examination or in his white blood cell count and inflammatory enzyme levels including erythrocyte sedimentation and C-reactive protein. Eight age-matched normal subjects (4 male, mean age, 26.6 years; range, 21–29 years) were enrolled in this study as control subjects. The patient's father and all normal subjects has provided informed consent for publication of the case, and the local ethics committee approved the study protocol.

### 2.1 Management and prognosis

When he showed hyperthermia during admission, we applied four types of management including external cooling methods (tepid massage and ice pack), hydration, and medication (propacetamol 1 g, intravenous injection).^[11–13]^ He revealed similar pattern of hyperthermia in terms of intensity and frequency during two month's admission at our hospital.

### 2.2 Diffusion tensor imaging

A multi-channel head coil on a 1.5 T Philips Gyroscan Intera (Philips, Best, Netherlands) with 32 gradients was used for the acquisition of DTI data. The patient's DTI data was acquired at 30 months after stroke onset. We acquired 67 contiguous slices parallel to the anterior commissure-posterior commissure line. DTI scanning parameters were as follows: acquisition matrix = 96 × 96; reconstructed to matrix = 192 × 192; field of view = 240 mm × 240 mm; repetition time = 10,398 ms; echo time = 72 ms; parallel imaging reduction factor = 2; echo-planar imaging factor = 59; b = 1000 s/mm^2^; number of excitations = 1; and slice thickness = 2.5 mm. Eddy current-induced image distortions were removed by using the affine multi-scale two-dimensional registration provided in the Oxford Centre for Functional MRI of Brain Software Library. DTI-Studio software (CMRM, Johns Hopkins Medical Institute, Baltimore, MD) was used for evaluation of the hypothalamus, which was identified by locating the anterior boundary of the optic tract and the posterior boundary of the mammillary body at the level of the upper midbrain (Fig. 1A).^[14]^ Fractional anisotropy (FA) and apparent diffusion coefficient (ADC) estimates were obtained for the hypothalamus. Regions of interest for the hypothalamus were localized by using the optic tract (anterior boundary), the mammillary body (posterior boundary), and the midline (medial boundary) at the level of the upper midbrain in the patient (C) and a representative normal subject.

## 3 Results

A summary of the DTI parameter results for the hypothalamus in the patient and the control groups is presented in Table 1. The FA and ADC value of the patient were lower and higher, respectively, by more than two standard deviations from the control values (Table 1).

**Table 1:** Diffusion tensor image parameters of the patient and control subjects.

	FA	ADC
Patient	0.16	2.25
Control subjects (n = 8)	0.25 (0.03)	1.09 (0.19)

Control values are presented as mean (SD). ADC = apparent diffusion coefficient, FA = fractional anisotropy.

## 4 Discussion

In this study, by assessing DTI parameters including FA and ADC, we evaluated the hypothalamus of a patient who showed neurogenic fever and leukomalactic lesion in the hypothalamus following intraventricular hemorrhage. The FA value indicates the degree of directionality of water diffusion, and the FA value reflect white matter integrity (e.g., loss of myelination, axon diameter, fiber density, or fiber organization).^[15]^ In contrast, the ADC value represents the magnitude of water diffusion in a tissue, which can increase with some forms of disease, particularly vasogenic edema or accumulation of cellular debris associated with neuronal injury.^[15]^ As a result, detection of a decrement in the FA value and an increment in the ADC value in this patient from those of the control subjects suggest the presence of a neural injury of the hypothalamus. Previous studies have suggested that injury to periventricular white matter by intraventricular hemorrhage could occur through mechanical (increased intracranial pressure or direct mass) or chemical (blood clot) mechanisms.^[16,17]^ Considering that the third ventricle is located in the hypothalamus, the hypothalamus appeared to be affected by a hematoma in this ventricle. As a result, it appears that the neurogenic fever of this patient was at least partly ascribed to injury of the hypothalamus.

Since the introduction of DTI, several studies have demonstrated relationships among various symptoms including narcolepsy, hypersomnia, cognitive fatigue, depression with the injury of the hypothalamus in multiple sclerosis, and traumatic brain injury.^[6–9]^ However, to the best of our knowledge, this is the first study to demonstrate that neurogenic fever can be due to injury of the hypothalamus in stroke patients. Nevertheless, this study is limited because it is a single case report; therefore, further prospective studies that include a large number of patients should be encouraged. In addition, some limitations of DTI should be considered.^[18]^ First, defining a region of interest for the measurement of DTI parameters in the hypothalamus can be difficult due to the poor resolution and small size of the hypothalamus. In addition, DTI parameters on the hypothalamus could be affected by a partial volume effect such as that associated with eddy currents due to cerebrospinal fluid.^[18]^

In conclusion, by using DTI, injury of the hypothalamus was demonstrated in a stroke patient with neurogenic fever. Our results suggest that evaluation of the hypothalamus by using DTI would be helpful in patients show unexplained fever following brain injury.

## Author contributions

Sung Ho Jang: Study concept and design, Manuscript development and writing, You Sung Seo: Acquisition and analysis of data, Study concept and design, Acquisition and analysis of data, Manuscript authorization

**Conceptualization:** sungho jang.

**Data curation:** YouSung Seo.

**Methodology:** YouSung Seo.

**Writing – original draft:** sungho jang.

**Writing – review & editing:** YouSung Seo.

## References

[1] Meier K Lee K. Neurogenic fever: Review of pathophysiology, evaluation, and management. J Intensive Care Med 2017;32:124–9. 26772198 10.1177/0885066615625194

[2] Agrawal A Timothy J Thapa A. Neurogenic fever. Singap Med J 2007;48:492–4. 17538744

[3] Thompson HJ Tkacs NC Saatman KE. Hyperthermia following traumatic brain injury: a critical evaluation. Neurobiol Dis 2003;12:163–73. 12742737 10.1016/s0969-9961(02)00030-x

[4] Afifi AK Bergman RA. Functional Neuroanatomy: Text and Atlas. New York, NY: Lange Medical Books/McGraw-Hill; 2005.

[5] Zhao ZD Yang WZ Gao CC. A hypothalamic circuit that controls body temperature (vol 114, pg 2042, 2017). Proc Natl Acad Sci USA 2017;114:E1755–1755. 28053227 10.1073/pnas.1616255114 PMC5338448

[6] Menzler K Belke M Unger MM. Dti reveals hypothalamic and brainstem white matter lesions in patients with idiopathic narcolepsy. Sleep Med 2012;13:736–42. 22541810 10.1016/j.sleep.2012.02.013

[7] Shen YJ Bai LJ Gao Y. Depressive symptoms in multiple sclerosis from an in vivo study with tbss. Biomed Res Int 2014;doi: 10.1155/2014/148465. 10.1155/2014/148465 PMC4024416 24877057

[8] Jang SH Yi JH Kim SH. Relation between injury of the hypothalamus and subjective excessive daytime sleepiness in patients with mild traumatic brain injury. J Neurol Neurosur Ps 2016;87:1260–U1134. 10.1136/jnnp-2016-313093 27044667

[9] Hanken K Eling P Kastrup A. Integrity of hypothalamic fibers and cognitive fatigue in multiple sclerosis. Mult Scler Relat Dis 2015;4:39–46. 10.1016/j.msard.2014.11.006 25787051

[10] Jang SH Kwon HG. Injury of the hypothalamus in patients with hypoxic-ischemic brain injury: a diffusion tensor imaging study. Am J Phys Med Rehab 2018;97:160–3. 10.1097/PHM.0000000000000813 28825946

[11] Goyal K Garg N Bithal P. Central fever: a challenging clinical entity in neurocritical care. J Neurocrit Care 2020;13:19–31.

[12] McGraw-Hill Professional, Fauci A Braunwald E Kasper D. Harrison's Principles of Internal Medicine. 17th ed. 2008;117–121.

[13] Thompson HJ Pinto-Martin J Bullock MR. Neurogenic fever after traumatic brain injury: an epidemiological study. JNNP 2003;74:614–9. 10.1136/jnnp.74.5.614 PMC1738450 12700304

[14] Duvernoy H Bourgouin P. The Human Brain: Surface, Three-Dimensional Sectional Anatomy with MRI, and Blood Supply. 2nd completely rev. and enl. Ed. New York, NY: Springer; 1999.

[15] Mori S Crain BJ Chacko VP. Three-dimensional tracking of axonal projections in the brain by magnetic resonance imaging. Ann Neurol 1999;45:265–9. 9989633 10.1002/1531-8249(199902)45:2<265::aid-ana21>3.0.co;2-3

[16] Chua CO Chahboune H Braun A. Consequences of intraventricular hemorrhage in a rabbit pup model. Stroke 2009;40:3369–77. 19661479 10.1161/STROKEAHA.109.549212 PMC2753705

[17] Yeo SS Choi BY Chang CH. Periventricular white matter injury by primary intraventricular hemorrhage: a diffusion tensor imaging study. Eur Neurol 2011;66:235–41. 21952179 10.1159/000330942

[18] Parker GJ Alexander DC. Probabilistic anatomical connectivity derived from the microscopic persistent angular structure of cerebral tissue. Philos Trans R Soc Lond Ser B Biol Sci 2005;360:893–902. 16087434 10.1098/rstb.2005.1639 PMC1854923

